# Schwann cells in the normal and pathological lung microenvironment

**DOI:** 10.3389/fmolb.2024.1365760

**Published:** 2024-04-04

**Authors:** Michael R. Shurin, Sarah E. Wheeler, Galina V. Shurin, Hua Zhong, Yan Zhou

**Affiliations:** ^1^ Department of Pathology, University of Pittsburgh Medical Center, Pittsburgh, PA, United States; ^2^ Department of Immunology, University of Pittsburgh Medical Center, Pittsburgh, PA, United States; ^3^ Department of Respiratory and Critical Care Medicine, Shanghai Chest Hospital, Shanghai Jiao Tong University School of Medicine, Shanghai, China

**Keywords:** Schwann cells, tumor innervation, metastasis, lung cancer, pulmonary diseases

## Abstract

The lungs are a key organ in the respiratory system. They are regulated by a complex network of nerves that control their development, structure, function, and response to various pathological stimuli. Accumulating evidence suggests the involvement of a neural mechanism in different pathophysiological conditions in the lungs and the development and progression of common respiratory diseases. Lung diseases are the chief source of death globally. For instance, lung cancer is the second most commonly diagnosed malignancy, after prostate cancer in men and breast cancer in women, and is the most lethal cancer worldwide. However, although airway nerves are accepted as a mechanistically and therapeutically important feature that demands appropriate emphasizing in the context of many respiratory diseases, significantly less is known about the role of the neuroglial cells in lung physiology and pathophysiology, including lung cancer. New data have uncovered some cellular and molecular mechanisms of how Schwann cells, as fundamental components of the peripheral nervous system, may regulate lung cancer cells’ survival, spreading, and invasiveness *in vitro* and *in vivo*. Schwann cells control the formation and maintenance of the lung cancer microenvironment and support metastasis formation. It was also reported that the number of lung cancer-associated Schwann cells correlates with patients’ survival. Different factors secreted by Schwann cells, including microRNA, are known to sharpen the lung cancer environment by regulating the tumor-neuro-immune axis. Further clinical and experimental studies are required to elucidate the detailed role of Schwann cells in creating and maintaining pulmonary tumor-neuro-immune axis, which will advance our understanding of the pathogenesis of lung cancer and may inform therapeutic hypotheses aiming neoplasms and metastases in the lung.

## Introduction

Respiratory illnesses are among the most widespread medical obstacles in the world and have a substantial worldwide health influence. For instance, chronic obstructive pulmonary disease (COPD) is the third leading cause of death affecting about 300 million people globally and contributing to more than 3 million deaths (Ruvuna and Sood, 2020). Asthma affects more than 340 million people and an additional 100 million may be affected by 2025 (Dharmage et al., 2019). An estimated ∼11 million people contract tuberculosis every year, and more than 1.5 million people die from tuberculosis annually making it the world’s leading cause of death from an infectious agent (Natarajan et al., 2020). Lung cancer is one of the most commonly diagnosed malignancies and is the most lethal cancer with an estimated annual 2 million new cases and ∼1.8 million deaths worldwide (Thai et al., 2021).

Lungs, as the foundational organs of the respiratory system, are innervated by the peripheral nervous system (PNS) and the vagus nerve that grants the lungs to perform their vital function in the respiratory system, which also includes the nose, oropharynx, larynx, trachea, bronchi, and bronchioles. The airways and lungs are heavily innervated with specific aiming of airway epithelium, smooth muscles, glands, and vasculature. Pulmonary innervation is responsible for dilating and constricting the airway via contraction of smooth muscles in bronchi and bronchioles, bronchial secretions from submucosal glands, vascular permeability, and blood flow. Sensory innervation of the visceral pleura is involved in stretch detection (Arreola-Ramírez et al., 2013).

One important aspect of the functioning of airway nerves is the involvement of a neural mechanism in different pathophysiological conditions in the lungs and the development or progression of common respiratory diseases. The well-established anatomical structure and integrity of airway innervation underscore the contribution of nerves to both the normal airway function and disease pathways that control airway tone, mucus secretion, inflammatory reaction, fibrosis, and other key characteristics (Canning and Spina, 2009; Kistemaker and Prakash, 2019). Accumulating evidence supports the concept that the respiratory neural network may be activated or dysregulated in COPD, asthma, chronic pulmonary fibrosis, pulmonary pain, chronic cough, lung infection, and other acute and chronic inflammatory respiratory diseases (van der Velden and Hulsmann, 1999; Undem and Kollarik, 2005; Undem and Nassenstein, 2009; Mazzone and Undem, 2016; Audrit et al., 2017; Kistemaker and Prakash, 2019; Yegen et al., 2023) For instance, airway nerves not only regulate airway physiology, they also express and secrete various pro- and anti-inflammatory molecules and may control attraction, homing, and activation of different subsets of immune cells (Verhein et al., 2009). Recent data revealed the essential role of local sympathetic innervations in negatively altering the lung’s innate immune reactions (Liu et al., 2020). Similarly, other data demonstrated significant activation of sympathetic nerve activity in patients with pulmonary artery hypertension, which may be associated with disease severity (Velez-Roa et al., 2004; Vaillancourt et al., 2017).

Thus, although airway nerves are accepted as a mechanistically and therapeutically important aspect that demands appropriate emphasizing in the context of many respiratory diseases, significantly less is done to reveal the role of PNS glial cells in lung physiology and pathophysiology.

### Schwann cells: Function and the role in different disorders

Schwann cells (SCs), discovered and described by German physiologist, Theodor Schwann, also known as neurolemmocytes, are primary non-neuronal cells, or neuroglial cells, of the PNS that support and regulate the viability and functions of peripheral neurons. Satellite cells, olfactory ensheathing cells, enteric glia, and glial cells at the endings of the sensory nerves are also members of the glial cells in the PNS. SCs differentiate from specific precursor cells in the neural crest and can be segregated into two types: myelinating and non-myelinating SCs. The myelinating SCs produce myelin for axon insulation and form cytoplasmic sheaths surrounding the axon of a single nerve. The non-myelinating Schwann cells line axons with their cytoplasm but do not produce myelin. SCs sustain peripheral nerve longevity under homeostatic conditions, independent of myelination (Eid et al., 2023).

SCs advance the saltatory transmission of nerve impulses as the velocity of electrical impulses along axons is up to ten times enhanced by the myelin sheath. SCs also account for supplying trophic support for neurons. SCs also play a crucial role in nerve regeneration (Balakrishnan et al., 2020). During damage of the PNS neurons, SCs first participate in direct or macrophage-mediated phagocytosis of broken or injured axons, a process known as Wallerian degeneration, associated with SC dedifferentiation, denervation, and proliferation. These repair SCs then form an endoneurial tunnel, which works as a guide track toward regenerating axons growing at a rate of about 1 mm per day. Importantly, axons die if SCs fail to interact with them (Jessen and Mirsky, 2016).

A growing body of evidence on the communication between SCs and immune cells implies SC’s contribution to inflammatory processes. Activated SCs can induce and control local immune reactions by presenting antigens and by expressing cytokines and chemokines that attract immune cells to the site of trauma or injury. Cross-talk with immune cells allows SCs to shape immune reactions that can be advantageous for inflammatory and autoimmune neuropathies (Meyer zu Hörste et al., 2008; Ydens et al., 2013). SCs are now known as antigen-presenting cells supporting neuron survival and preserving axons (Hartlehnert et al., 2017). For instance, recent data characterizing repair SCs revealed high levels of expression of MHC class II molecules and co-stimulatory and co-inhibitory molecules, including CD40, CD80, CD86 CD58 (or LFA-3), and CD276 (or B7-H3) (Berner et al., 2022). The ability of these SCs to produce both pro- and anti-inflammatory cytokines and chemokines, regulate PD-L1-mediated T cell inhibition, and control the activation state of T cells suggest their functional similarities with professional antigen-presenting cells and immune regulators of T cell responses during nerve repair process (Berner et al., 2022). SCs interaction with peripheral nerve resident macrophages may be responsible for cancer-induced pain, at least in some experimental systems (De Logu et al., 2021).

Because SCs have been proven to play an important role in neurotransmission, axon de/regeneration, protection of nerves in the PNS, interaction with immune cells, and shaping the tumor microenvironment, they are a cluster of glial cells of great clinical significance. Different disorders are related to the deterioration, damage, hyperactivation, or malfunction of SCs including demyelinating neuropathies (e.g., inherited, inflammatory, autoimmune), spinal cord injury, neuropathic pain, infections, and cancer. Understanding cellular and molecular mechanisms of SC-associated pathophysiology and progression of these diseases is well justified for improving disease diagnostics, predicting patient outcomes, and developing effective therapeutic approaches. Furthermore, targeting pathological SCs or adoptive transfer of normal or modified SCs is a novel therapeutic modality that should be available soon.

### Innervation of the lung in normal conditions and different diseases

**FIGURE 1:**
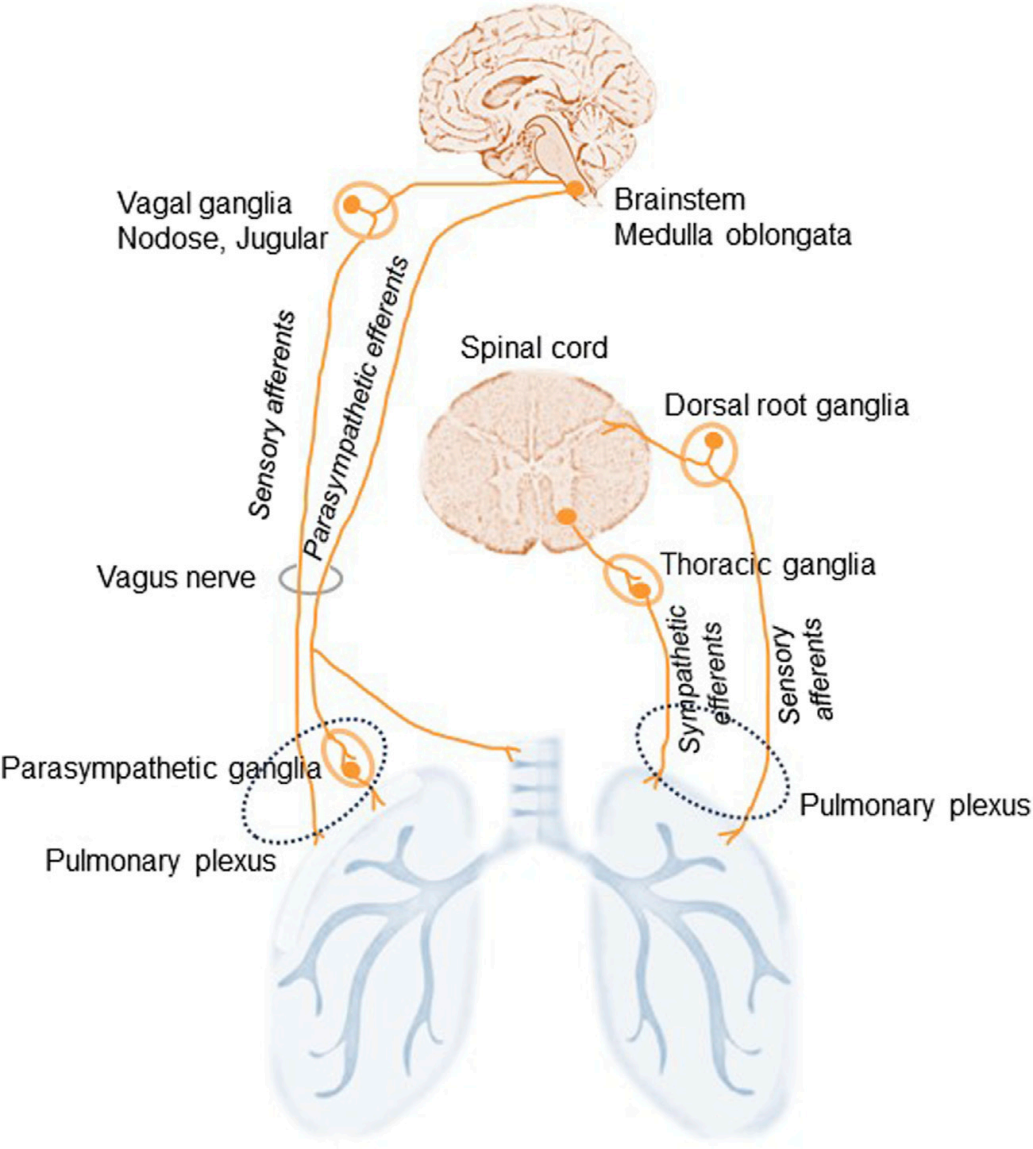
Schematic illustration of lung innervation. The lungs are innervated by the sympathetic, parasympathetic, and sensory branches of the peripheral nervous system. The vagus nerves supply parasympathetic, originated in the medulla oblongata, and sensory, via the nodose and jugular ganglia, nerve fibers to the airways. Additional sensory nerves travel from the dorsal root ganglia of the spinal cord and travel with spinal sympathetic nerves. Sympathetic efferents of the thoracic ganglia of the sympathetic trunk originate in the ventral horn of the spinal cord and run via the pulmonary plexus. Sympathetic nerves: adrenergic. Parasympathetic nerve: cholinergic.

The lungs are highly innervated by the sympathetic, parasympathetic, and sensory branches of the PNS (Downing and Lee, 1980). They are innervated from the pulmonary plexus in the root of each lung and the phrenic nerve, which comes from C3,4,5 cervical nerve roots. Branches from the vagus nerve and sympathetic branches from the cervical cardiac nerves (sympathetic trunk) merge and establish the pulmonary plexus (Figure 1). The plexus is then subdivided into anterior and posterior branches matching the hilum of the lung supplying the bronchi and the visceral pleura (Belvisi, 2002). The vagus nerves, which provide all parasympathetic and most of the sensory nerve fibers to the airways, divide into the superior laryngeal and recurrent laryngeal nerves, which carry sensory fibers and preganglionic parasympathetic fibers to the trachea and main bronchi. Smaller branches of the vagus then supply the rest of the airways. Preganglionic autonomic fibers are myelinated, whereas the postganglionic part is usually unmyelinated. Sympathetic nerves innervating the lung begin in the upper thoracic sections of the spinal cord and the sympathetic ganglia synapse innervating bronchial blood vessels and submucosal glands (Figure 1). The sympathetic nervous system regulates bronchodilation and mucous secretion, while the parasympathetic system regulates bronchoconstriction.

Vagal sensory neurons establish the main afferent stream to the airways and lungs: approximately 20% of afferent vagus nerves terminate in the airways and lungs (Mazzone and Undem, 2016). Nociceptive afferent nerve endings localized in the lung parenchyma and near the airways respond to different noxious stimuli including irritants, allergens, and pathogens. Some more minor sensory innervation originates from the T1-T6 dorsal root ganglia and these fibers run with spinal sympathetic nerves (Lee and Yu, 2014).

Thus, three types of nerve fibers link the lungs to the autonomic part of the PNS (Kistemaker and Prakash, 2019). Two efferent fibers: the parasympathetic fibers in the vagus nerve provide motor stimuli to the airways and lungs inducing bronchiolar muscle contraction, vasodilatation, and gland secretion; and the sympathetic fibers responsible for relaxation of bronchial muscles, vasoconstriction, and suppression of glandular secretion. The third type of nerve fibers in the lung are afferent neurons that are subdivided into A- and C-fibers and the so-called cough receptors (Audrit et al., 2017). The afferent A-fibers in the vagus nerve connect stretch receptors in the airways and the lungs with the pulmonary plexus. The axons of these receptors are myelinated and have high conduction velocities. Another class of airway sensory fiber is the unmyelinated bronchial or pulmonary C-fiber (McGarvey, 2015). Pulmonary C-fiber endings are found in the lung parenchyma, while bronchial C-fibers are seen within the airway mucosa (Canning and Spina, 2009; Su et al., 2021).

Clinical data suggest that nerves in the lungs can be affected by different diseases; for instance, lung cancer can spread to the nerve trunks. Respiratory dysfunctions resulting from neurological diseases are well described (Michael et al., 1999). New studies also highlight the roles of lung-innervating neurons in controlling immune cell activity in asthma, rhinitis, chronic obstructive pulmonary disorder, and lung infections (Blake et al., 2019; De Virgiliis and Di Giovanni, 2020). For instance, vagotomy has been reported to worsen lung infection, inflammation, and injury implying that the pulmonary parasympathetic inflammatory reflex can control the extent of lung infection and inflammation (Huang et al., 2019). Interestingly, PNS nerves have been suggested as an important neuroanatomical component of the bi-directorial brain-lung axis (Li et al., 2023). Functional communication between the lung and the brain is associated with many disorders including lung diseases and CNS diseases.

Lung innervation was also investigated in resected lung specimens from lung adenocarcinoma patients and the results demonstrated a negative correlation between the density of neural fiber expression and recurrence-free survival in patients with lung adenocarcinoma (Shao et al., 2016). Interestingly, sympathetic nerve fiber density was higher in the peritumor area, while parasympathetic nerve fibers were located in the tumor bed. Thus, PNS nerve densities associated with lung adenocarcinoma are negatively correlated with disease prognosis. These data correlate with the report that a comprehensive suppression of sympathetic activity may decrease the risk of developing primary pulmonary tumors in smokers and nicotine addicts (McCarty et al., 2015). Other data revealed that non-small cell lung cancer (NSCLC) patients who used beta-blockers have better disease-free survival and lower rate of metastasis than patients on conventional cancer therapy (Coelho et al., 2020). In fact, beta-blocker propranolol was shown to significantly attenuate the proliferation of lung cancer cell lines (Al-Wadei et al., 2012). Similarly, the blockage of muscarinic cholinergic signaling has been demonstrated to inhibit small cell lung cancer (SCLC) and NSCLC growth in different model systems (Zhao et al., 2015; Spindel, 2016). Similar clinical and experimental results were also reported for different types of solid tumors (Hutchings et al., 2020).

### Glial cells in the lungs: Identification, characterization and significance

Although the lung hosts both myelinating and non-myelinating SCs, the latter correspond to the amplest SC subpopulation in the lung (Heredia et al., 2023) since almost 90% of nerves in the lung are unmyelinated (Jammes et al., 1982). Non-myelinating SCs in the lung were first described in 1957 (Agostoni et al., 1957) (Table 1), and electron microscopy and immunohistochemical analyses of lung innervation reliably reveal a spatial location of SCs and the axons of pulmonary nerves (Hung et al., 1972; Sparrow et al., 1999; Suarez-Mier and Buckwalter, 2015; Heredia et al., 2023). For instance, S-100-positive glial cells were described as cells surrounding peripheral nerves in the lung in man and three other mammalian species (Sheppard et al., 1983). Description of the morphology, tissue distribution, and connection of S-100-positive glial cells with pulmonary nerves was also confirmed in transgenic mice that express a green fluorescent protein (GFP) in the lung under the control of glial fibrillary acidic protein (GFAP) promoter, a marker of non-myelinating SCs (Suarez-Mier and Buckwalter, 2015). Glia-ensheathed nerves create a netting that surrounds large and small bronchi (Suarez-Mier and Buckwalter, 2015). Glia of non-myelinating SCs have been also found ensheathing blood vessel nerves (Table 1).

**TABLE 1:** Schwann cells in the lung microenvironment.

Lung-associated Schwann cells	Main findings	References
Localization and characterization of SCs in the pulmonary tissues		
Identification of SCs in the lung	Non-myelinating SCs have been described in the lung	Agostoni et al. (1957)
Description of SCs in the lung by electron microscopy and immunohistochemistry	A spatial location of SCs and the axons of pulmonary nerves	Hung et al. (1972), Sheppard et al. (1983), Sparrow et al. (1999), Suarez-Mier and Buckwalter (2015), Heredia et al. (2023)
Depiction of the morphology and tissue distribution of lung-associated SCs	Description of glia-ensheathed nerves surrounding large and small bronchi and blood vessel	Suarez-Mier and Buckwalter (2015)
SC precursors and immature SCs in the lung	Documented expression of glia markers during respiratory neurogenesis	Sparrow et al. (1999), Burns et al. (2008), Aven and Ai (2013), Dyachuk et al. (2014), Sountoulidis et al. (2023)
Schwann cells in lung cancer		
Recognition of SC in lung cancer tissue	Demonstration of the presence of SCs	Silva et al. (2019), Cao et al. (2022)
SC identification in NSCLC tissues	Increased numbers of detectable SCs positively correlated with a worse patient prognosis	Zhou et al. (2019)
Protumorigenic activity of SCs in lung cancer	SCs could regulate the motility and invasiveness of tumor cells *in vitro* and the development of metastases *in vivo* in animal models	Zhou et al. (2018), Zhou et al. (2020)
SC identification in SCLC tissues	Tumor-associated SCs upregulated longevity, proliferation, migration, and invasiveness of tumor cells	Cao et al. (2022)
Protumorigenic activity of SCs in NSCLC *in vitro* and *in vivo*	MicroRNA from SC-derived exosomes may promote NSCLC progression	Zhou et al. (2024)

NSCLC, non-small cell lung cancer; SCs, Schwann cells; SCLC, small cell lung cancer.

During lung development, in addition to the vagal neurons, neural crest cells also migrate to develop parasympathetic ganglia constituting both neurons and glial cells (Burns et al., 2008). Expression of glia markers was documented during respiratory neurogenesis (Aven and Ai, 2013). Neural crest-derived SOX10^+^PHOX2B^+^ SC precursors enter the lung, travel toward ganglionic positions, and differentiate into parasympathetic neurons and immature SCs in the bronchial interstitium (Dyachuk et al., 2014; Sountoulidis et al., 2023). In humans, an association of ganglia and nerve trunks with SCs in the developing airways was demonstrated by the middle of the first trimester (Sparrow et al., 1999).

The presence of SCs in the lungs is also demonstrated by the identification of pulmonary schwannomas - nerve sheath tumors comprised of neoplastic SCs. Schwannomas, also known as neurilemmomas or neurinomas of Verocay, are seen as encapsulated benign tumors expressing SC differentiation morphology and markers, including S100 and SOX10 protein, that develop from myelinated peripheral nerves (Belakhoua and Rodriguez, 2021). Because primary lung nerve cell tumors are very rare, accounting for ∼0.2% of all lung neoplasms, only a few reports describe cases of pulmonary schwannoma (Roviaro et al., 1983; Ohtsuka et al., 2005; Abbas et al., 2023).

Recent data suggest that non-myelinating SCs in the lung not only provide axon support and protection but are also actively involved in coordinating lung inflammation (Heredia et al., 2023). Remarkably, characterization of mouse lungs myelinating and non-myelinating SC transcriptome using single-cell RNA sequencing revealed the presence of two non-myelinating SC populations that participate in pathogen recognition. Comprehensive analysis of proinflammatory response of lung SCs both *in vitro* and *in vivo* uncovered that lung non-myelinating SCs, but not myelinating SCs, were actively involved in the innate immune response in the lung and lung inflammation (Heredia et al., 2023).

Furthermore, during lung cancer development and progression, SCs in the local lung microenvironment may play a quite different and specific role by interacting with both the malignant cells and infiltrating effector and regulatory immune cells.

### Schwann cells in the lung cancer milieu: Molecular mechanisms of tumor promotion

SCs were detected in the lung tumors and in the tumor surrounding area both in experimental animals and human specimens (Silva et al., 2019; Cao et al., 2022). Furthermore, the retrospective analysis of SC identification in NSCLC tissues comprising 90 cases of adenocarcinoma and 51 cases of squamous cell carcinoma revealed higher levels of SCs in cancerous tissues compared to adjacent tissues (Zhou et al., 2019). Importantly, increased homing of detectable SCs positively correlated with a worse patient prognosis. Furthermore, it was also observed that advanced stages of lung cancer exhibited the highest levels of SC detection (Zhou et al., 2019). Although the identification of SCs in different solid tumors and their role in the development and metastasis formation in different types of cancer have been repeatedly reported and reviewed (Bunimovich et al., 2017; Deborde and Wong, 2022; Sun et al., 2022; Yurteri et al., 2022; Xue et al., 2023), data describing the functional significance of tumor-associated SCs (TA-SCs) in lung cancer are scarce (Table 1).

**FIGURE 2:**
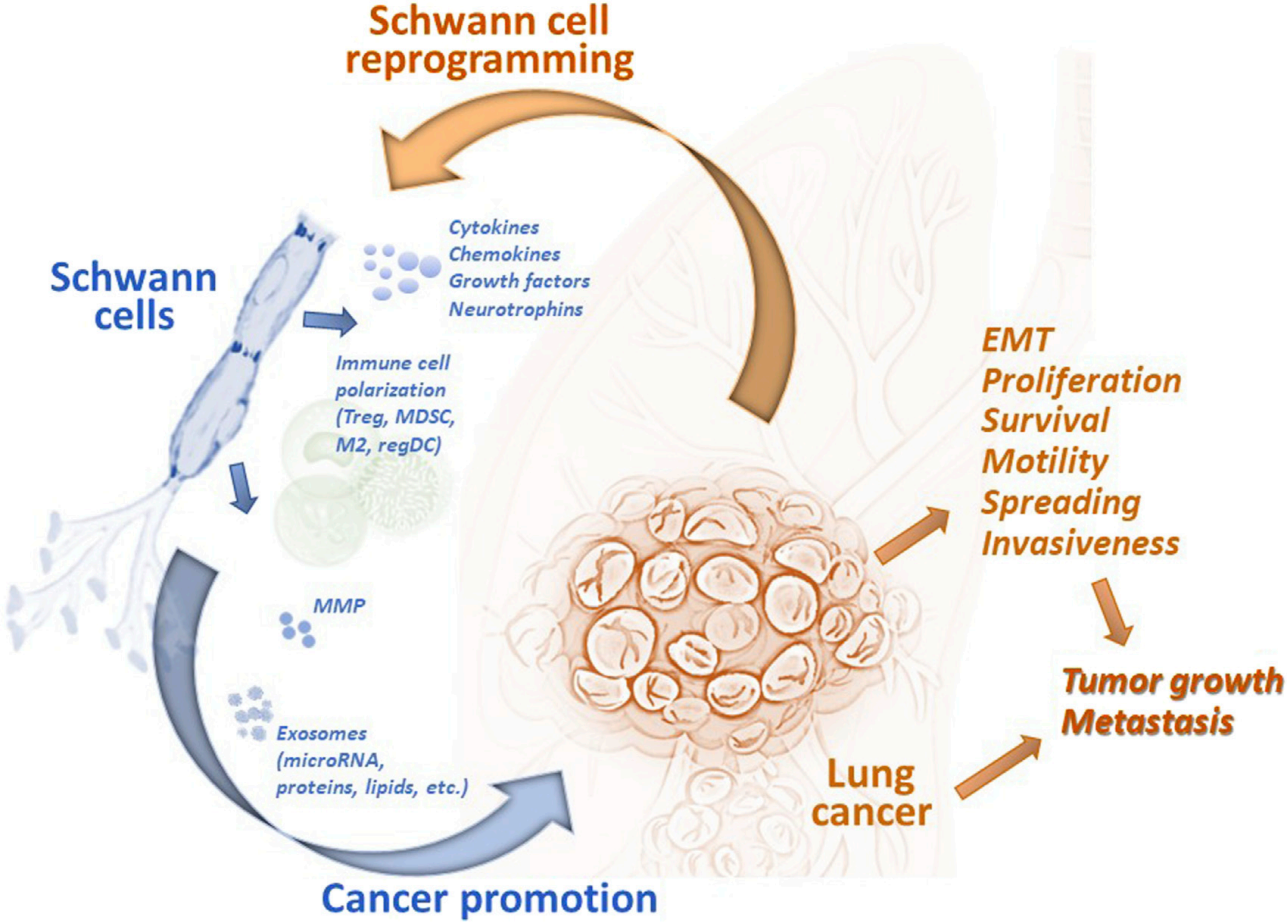
Tumor-associated Schwann cells can promote development and progression of lung cancer. Schwann cells have been identified in the lung tumor specimens, and their number has been shown to correlated with the prognosis of disease. Tumor-associated remodeled Schwann cells can be reprogrammed by tumor-derived factors to the repair-like phenotype and activated to express and release cytokines, chemokines, growth and neurotrophic factors, matrix metalloproteinases, and exosomes. These and other factors modulate the tumor microenvironment by (i) attracting and polarizing immune cells to the regulatory (immunosuppressive) phenotype, (ii) remodeling the extracellular matrix, and (iii) directly altering the functional activity of malignant cells. Schwann cell-derived chemokines can upregulated EMT in lung cancer cells and stimulate their transmigratory potential. microRNA from Schwann cell exosomes promote proliferation, motility, and invasiveness of lung cancer cells by blocking specific mRNA in malignant cells. Altogether, tumor-associated Schwann cells can promote lung tumor growth *in vivo* and support formation of distant metastases. EMT, epithelial-mesenchymal transition; M2, alternatively activated tumor-associated macrophages; MDSC, myeloid-derived suppressor cells; MMP, matrix metalloproteinases; regDC, regulatory dendritic cells; Treg, regulatory T cells.

Earlier reports utilizing experimental animal models showed that SCs could regulate the motility and invasiveness of lung cancer cells *in vitro* and the development of metastases *in vivo*. SCs have been shown to endorse the epithelial-mesenchymal transition (EMT) and transmigration of lung cancer cells via CXCR2 by releasing chemokine CXCL5, augmenting expression of Snail and Twist, functional markers of EMT, and activating the PI3K/AKT/GSK-3β/Snail-Twist signaling pathway in lung cancer cells (Zhou et al., 2018). Furthermore, treatment of tumor cells with SC conditioning medium before the tumor cell inoculation resulted in a significant acceleration of metastasis formation in the regional lymph nodes in mice (Zhou et al., 2018). However, after knocking down CXCL5 signaling, a significant reduction in metastasis formation was observed. These data confirmed a new role of the PNS and specifically SCs in the functional organization of the lung tumor microenvironment and tumor progression (Figure 2).

Cellular mechanisms of the pro-tumorigenic activity of SC include both the direct effect on malignant cells and the modulation of infiltrating immune cells in the tumor immunoenvironment (Figure 2). Attraction and polarization of conventional dendritic cells, immature myeloid cells, and T cells into immunosuppressive regulatory dendritic cells, myeloid-derived suppressor cells (MDSC), and T regulatory (Treg) cells, respectively, have been reported in different tumor models (Martyn et al., 2019; Zhang et al., 2020; Shurin et al., 2022). For lung cancer, bioinformatic analysis of the transcriptome of SCs demonstrated the secretion of high levels of CCL2, CXCL5, CXCL12, and CXCL8, and functional data suggested that CCL2 promoted the M2 polarization of macrophages (Zhou et al., 2020). Furthermore, SC-polarized M2 macrophages augmented the proliferation of lung cancer cells, demonstrating another functional path of the tumor-PNS/glia-immune axis.

According to the genome-scale biomolecular network analysis of SCs in lung adenocarcinoma and lung squamous cell carcinoma, pathways of SC dedifferentiation were upregulated while the expression of genes representing SC migration inhibition system was decreased. Interestingly, miRNAs targeting these pathways were also significantly altered (Silva et al., 2019). These results suggest that identified intratumoral SC-associated molecules may be used as systems biomarkers for screening in perineural invasion of lung cancer or for potential therapeutic purposes (Silva et al., 2019). Importantly, new data focusing on the ability of human TA-SCs to regulate the functional activity of human SCLC cell lines *in vitro* revealed that TA-SCs upregulated longevity, proliferation, migration, and invasiveness of tumor cells (Cao et al., 2022). *In vivo* studies confirmed these findings by demonstrating that SCs promote the growth of SCLC in mice. Further analysis of human TA-SCs revealed that they exhibit a repair-like phenotype, confirming results reported for SC analysis in melanoma specimens (Shurin et al., 2019). This allows distinguishing TA-SCs as so-called tumor-associated repair or repair-like SCs. Investigation of differentially expressed genes in lung cancer-activated repair-like SCs allowed the construction of unique networks of messenger-, micro-, and long non-coding RNA (mRNA-miRNA-lncRNA) that brought new insights into our understanding of potential molecular mechanisms of pro-tumorigenic activity of lung cancer-associated SCs. Furthermore, using the gene ontology (GO) enrichment annotations for the analysis of differentially expressed mRNAs in the SC-treated SCLC cells revealed a series of significant alterations that were categorized in all three common groups: biological process, cellular component, and molecular function (Cao et al., 2022). These new findings identified a new functional phenotype of SCs in cancer and brought new development into a better characterization of the nervous system-tumor crosstalk.

New data provide an interesting mechanistic explanation of how SCs can interact with malignant cells in the lungs. It is generally accepted that SCs utilize extracellular vesicles or exosomes to build and maintain the nerve microenvironment. Exosomes, as a means of intercellular communication, are extracellular vesicles that are consistently or inducibly released by many cell types to transfer protein, lipids, nucleic acids, glycoconjugates, membrane-bound molecules, and other biologically active factors to surrounding or distant cells and tissues (Edgar, 2016). This pathway of intercellular vesicle traffic plays important roles in many aspects of SC’s ability to transfer distinct cargoes that employ either neuroprotective and neuroregenerative or pathogenic influence on the recipient cells. For instance, the role of SC-derived exosomes in promoting axonal regeneration has been experimentally proven (Lopez-Verrilli et al., 2013; López-Leal et al., 2020; Wong et al., 2022). Mutations in the exosome biogenesis-related proteins causing a reduced number of exosomes and excretion of exosomal proteins in SCs were implicated in the molecular pathogenesis of Charcot-Marie-Tooth disease, a common hereditary neurological disorder of the PNS (Zhu et al., 2013). Interestingly, apart from different neuropathies, the potential contribution of SC-derived exosomes in carcinogenesis has also been suggested (Wong et al., 2022). A case study of schwannomatosis revealed a promoting effect on prostate-specific antigen secretion and a growth-accelerating outcome on a cognate schwannoma after resection of a prominent schwannoma (Chignon-Sicard et al., 2019). This was explained by exosomes secreted from schwannoma cells and tumor-adjacent resident SCs that targeted prostate cells and distant schwannoma cells. In addition, the possibility that SC’s exosomes can mediate the formation of a pre-metastatic niche has also been speculated (Wong et al., 2022). However, direct evidence of tumor cell stimulation by SC-derived exosomes has only been published recently.

The results of a new study, using the *in vitro* and xenogeneic *in vivo* models of NSCLC, revealed that the promotion of tumor cell proliferation, survival, motility, and transmigration by human SC-conditioned medium was due to SC-derived exosomes (Zhou et al., 2024). Evaluation of the expression and functional activity of SC exosomal microRNA demonstrated that hsa-miRNA-21-5P is responsible for upregulation of NSCLC cell longevity and motility. In fact, the necessity of exosome secretion from SCs was verified by knocking down Rab small GTPases Rab27A and Rab27B, which are known to control exosome secretion in human cells (Ostrowski et al., 2010) by shRNA-based targeting. These *in vitro* results were further confirmed by assessing the function of tumor cells transduced with miRNA-21-5p mimic and inhibitor. Additional analysis of tumor cells for intracellular signaling affected by miRNA-21 revealed that reversion-inducing cysteine-rich protein with Kazal motifs (RECK), a matrix metalloproteinase (MMP) inhibitor, not only blocked the interaction with exosomal miR-21-5p but completely prevented activation of tumor cells when transfected into RECK knockout tumor cells in a mutant form (Zhou et al., 2024).

The *in vivo* analysis of exosomal microRNA from SCs utilizing Cell Line-derived Tumor Xenograft models demonstrated that the presence of exosomal miRNA-21-5p was required for SC exosome-mediated stimulation of NSCLC growth *in vivo*. Importantly, by using EGFP-transfected tumor cells, it was also reported that SC exosomes significantly promoted the metastatic activity of tumor cells *in vivo* when miRNA-21-5p activity was not inhibited (Zhou et al., 2024). Finally, the confirmation of the co-expression of S100B positive SCs and miRNA-21 in human lung cancer tissues and the demonstration of an association between hsa-miRNA-21-5P expression levels and poor prognosis for NSCLC patients supported the clinical significance and high translational potential of these new findings.

Thus, these data demonstrate that exosomal microRNAs released from TA-SCs may potentiate proliferation, migration, and invasiveness of lung cancer cells, and upregulate tumor growth and metastasis formation of lung cancer. Together with other findings showing that SC-derived factors support lung cancer progression, these data provide a prospect to assess novel cellular and molecular targets for lung cancer therapy.

## Conclusion

Our recognition of the relevance of SC activity and lung cancer is still in its infancy although the important role of SCs in other cancer types has already been demonstrated (Deborde and Wong, 2017; Martyn et al., 2019; Shurin et al., 2021; Deborde and Wong, 2022; Yurteri et al., 2022). Analysis of SC biology in prostate, pancreatic, cervical, oral, colon tumors, melanoma, and other cancer types revealed their involvement in perineural invasion, cancer pain, and tumor cell proliferation, dispersion, invasion, and metastasizing (Demir et al., 2014; Deborde et al., 2016; Shan et al., 2016; Shurin et al., 2019; Huang et al., 2020; Salvo et al., 2021; Santi et al., 2022). It was also shown that the proregenerative state of tumor-activated SCs is important for the stimulation of tumor development, progression, and metastasis (Shurin et al., 2020; Pascual et al., 2021; Deborde and Wong, 2022). Nevertheless, there are convincing findings concerning the significance of cytokines, chemokines, and exosomes in SC’s ability to control lung cancer development, progression, and metastasis formation directly or via modulating the tumor immunoenvironment. These data include reports showing that lung cancer-associated or activated/polarized SCs: i) are strongly associated with a poor prognosis in patients with lung cancer, ii) promote longevity, motility, and spreading of different lung cancer cell lines *in vitro*, iii) endorse EMT of lung cancer cells, iv) accelerate lung cancer cell growth and metastasis formation *in vivo*, v) express cytokines and chemokines that upregulate polarization of regulatory immune cells, vi) release exosomes containing microRNA that modulate various functions of lung cancer cells, and vii) regulate lncRNA-miRNA-mRNA competitive endogenous RNA (ceRNA) networks that play critical roles in development of lung neoplasms.

Collectively, available data suggest that SCs regulate lung cancer progression, indicating that manipulation of resident and, potentially, adoptively transferred SCs may provide a useful tool to improve lung cancer patients’ outcomes. Future studies in this field will permit a better understanding of SC-controlling pathways in the lung cancer milieu in search of new multimodal diagnostic and therapeutic approaches in patients with SCLC and NSCLC. New strategies targeting repair-like TA-SCs or SC-derived exosomes as therapeutics and the development of diagnostic panels evaluating TA-SC-associated ceRNA networks as new biomarkers will contribute to improving outcomes of patients with lung cancer.
